# Hyperthermia Disturbs and Delays Spontaneous Differentiation of Human Embryoid Bodies

**DOI:** 10.3390/biomedicines8060176

**Published:** 2020-06-26

**Authors:** Ji Hyun Kwon, Hyun Kyu Kim, Tae Won Ha, Jeong Suk Im, Byung Hoo Song, Ki Sung Hong, Jae Sang Oh, Jaeseok Han, Man Ryul Lee

**Affiliations:** 1 Soonchunhyang Institute of Medi-bio Science (SIMS), Soon Chun Hyang University, Cheonan 31151, Korea; jihyun5861@daum.net (J.H.K.); hyunkyu8505@naver.com (H.K.K.); htw5200@gmail.com (T.W.H.); vjvmf625@naver.com (J.S.I.); thdqudgn@naver.com (B.H.S.); 2 Department of Medicine, Konkuk University School of Medicine and Mirae Cell Bio Co. LTD., Seoul 05029, Korea; stemness@gmail.com; 3 Department of Neurosurgery, College of Medicine, Soonchunhyang University, Cheonan Hospital, Cheonan 31151, Korea

**Keywords:** hyperthermia, embryogenesis, human embryonic stem cells, endoplasmic reticulum, stress response

## Abstract

Various types of stress stimuli have been shown to threaten the normal development of embryos during embryogenesis. Prolonged heat exposure is the most common stressor that poses a threat to embryo development. Despite the extensive investigation of heat stress control mechanisms in the cytosol, the endoplasmic reticulum (ER) heat stress response remains unclear. In this study, we used human embryonic stem cells (hESCs) to examine the effect of heat stress on early embryonic development, specifically alterations in the ER stress response. In a hyperthermic (42 °C) culture, ER stress response genes involved in hESC differentiation were induced within 1 h of exposure, which resulted in disturbed and delayed differentiation. In addition, hyperthermia increased the expression levels of activating transcription factor 4 (ATF4) and C/EBP homologous protein (CHOP) genes, which are associated with the protein kinase RNA-like endoplasmic reticulum kinase (PERK) signaling pathway. Furthermore, we demonstrated that tauroursodeoxycholic acid, a chemical chaperone, mitigated the delayed differentiation under hyperthermia. Our study identified novel gene markers in response to hyperthermia-induced ER stress on hESCs, thereby providing further insight into the mechanisms that regulate human embryogenesis.

## 1. Introduction

Hyperthermia or fever refers to a temporary rise in body temperature as a complex, systemic physiological response to infection or injury, serving as a defense mechanism to cellular stress [1]. Cells regulate their transcription and translation mechanisms in response to an increase in body temperature for various reasons. In addition, hyperthermia modifies the physical structure of intracellular enzymes by suppressing protein folding and denaturing mature proteins, resulting in the accumulation of misfolded or unfolded proteins that ultimately disturbs protein homeostasis in cells [2]. Upon sudden exposure to harsh external environmental conditions such as heat shock, cells initiate a self-defense mechanism known as the “heat stress response” including the heat shock response (HSR), which is mediated by the transcription factor heat shock factor 1 (HSF1) in the cytosol [3]. Under normal conditions, HSF1 constitutes a complex comprising the chaperone heat shock protein 90 (HSP90) that maintains HSF1 in an inactive state [4]. However, when exposed to heat, the accumulated unfolded proteins compete with HSF1 for the binding site of HSP90, resulting in the release of HSF1 from the HSP90 complex. In turn, free active HSF1 induces the transcription of heat shock genes, which encode cytosolic chaperones such as HSPA1A (HSP70) and DNAJB1 (HSP40) [5]. During heat stress, these HSPs function as chaperones in the cytosol, combine with unfolded proteins, and contribute to maintaining cellular homeostasis by preserving these proteins until the heat stress is alleviated, or by degrading denatured proteins to minimize the accumulation of abnormal proteins [6,7,8].

In contrast to the HSP regulation mechanism in the cytosol, the endoplasmic reticulum (ER) also regulates the unfolded proteins accumulated by heat stress with a distinct mechanism. Three signaling pathways in the ER membrane are involved in controlling denatured proteins accumulated in the ER: pancreatic ER kinase (PERK), inositol-requiring 1a (IRE-1a), and activation transcription factor 6 (ATF6) [9]. The accumulation of denatured proteins leads to the release of binding immunoglobulin proteins (BiP) from ER membrane proteins, which weakens the global protein synthesis by activating downstream genes [10]. With this series of unfolded protein response (UPR) processes, cells can remove denatured proteins and maintain their normal state. However, few studies have detailed which genes are affected during heat-induced ER stress and the subsequent impact on cell differentiation during development.

Hyperthermia has a negative effect on mammals by affecting temperature-dependent mechanisms that regulate the progressive development from the fertilized egg stage to the embryonic, prenatal, and postnatal stages, as well as the physiological processes, cell division, proliferation, and differentiation; thus, it influences organ formation and development [11]. Hyperthermia causes extensive structural and functional defects in the body, including the central nervous system, and leads to severe damage to cells and tissues [12]. In particular, as a developing embryo or fetus has limited capacity to control its own temperature, exposure to extreme heat stress has a direct effect on cell migration, proliferation, differentiation, and apoptosis, and can potentially lead to abnormal embryonic development, delayed growth, or prenatal death. Studies have reported that high temperature led to defects in neural tube development and craniofacial development, including clefts, the axial and appendicular skeleton, body wall, and teeth, along with the heart and almost all other organs [13]. Usually, higher temperatures and longer exposures to high temperatures increase the occurrence of such abnormalities and the risk of miscarriage.

Although previous experiments have observed the occurrence of abnormalities caused by hyperthermia in almost all experimental animal species, a few of these findings have been confirmed in humans. The main reason for the lack of studies on human embryogenesis under heat stress is that the normal body temperature of an experimental animal is not the same as that of a human, which impairs the translation of the results from animal studies to humans [14]. Furthermore, most experimental animals are smaller than humans, have a different basal metabolic rate, and the levels of heat sensitivity vary across different species [1]. In particular, studies examining the effect of hyperthermia on early human embryogenesis are hindered by the lack of a suitable in vitro model of an early human embryo. It is generally recognized that faster heat damage to a developing embryo has a greater effect on malformation or embryogenesis; however, most of these studies have been limited to animal models, which makes it difficult to directly apply these results to human embryogenesis.

To overcome these limitations, in the present study, we used human embryonic stem cells (hESCs) to examine the effects of heat stress on early human embryogenesis. hESCs originating from the inner cell mass of the blastocyst stage at 4–5 days post-fertilization represent a crucial in vitro cell resource for identifying the genetic factors contributing to early embryogenesis and organogenesis [15]. With embryoid body (EB) formation, hESCs can replicate three-germ layer differentiation in vitro, which has contributed to the identification of various mechanisms involved in early embryogenesis and highly organized cell differentiation systems. By mimicking early human embryo differentiation using hESCs as an in vitro model, we aimed to reveal the defects caused by hyperthermic culture conditions (42 °C) during differentiation in terms of the ER stress response mechanism.

## 2. Experimental Section

### 2.1. Culture and Maintenance of hESCs

The WA09 hESC line (H9; WiCell Research Institute, WiCell, Madison, WI, USA) was maintained in the undifferentiated state using vitronectin and TeSR-E8 medium (Stem Cell Technologies). The cells were plated in vitronectin-coated dishes and incubated at 37 °C with 5% CO_2_. The medium was changed every 24 h. For passaging, the cells were enzymatically detached with TrypLE Express (Gibco, Thermo Fisher Scientific, Waltham, MA, USA) and transferred to a new coated dish every 5 days. Passages were made at a 1:4 or 1:5 split ratio.

### 2.2. EB Generation to Induce Spontaneous In Vitro Differentiation

For EB generation, clumps of undifferentiated hESCs were mechanically detached with a glass pipette, seeded onto non-adhesive bacterial dishes in a differentiation medium without basic fibroblast growth factor, and allowed to spontaneously form into EB as an initial differentiation method [16]. The differentiation medium was changed daily and consisted of Dulbecco’s modified Eagle medium (DMEM)/F12 (Invitrogen, Gibco, Carlsbad, CA, USA) containing 20% KnockOut Serum Replacement (Invitrogen, Gibco, Carlsbad, CA, USA), 1 mM glutamine, 1% nonessential amino acids, 0.1% penicillin/streptomycin, and 0.1 mM beta-mercaptoethanol.

### 2.3. Hyperthermia and Chemical Chaperone Treatment

To impose heat stress on the differentiating human EB (hEB), EBs were first generated as described above and incubated at 37 °C with 5% CO_2_ for 2 days. Previous studies suggested 42 °C as the appropriate temperature for these experiments [17]. To determine the optimal heat exposure time, the hEBs were exposed to 42 °C, which is generally not enough to cause cytotoxicity [18], in the 5% CO_2_ incubator for different periods of time. After exposure to hyperthermic conditions, the hEBs were further cultured at the physiological temperature of 37 °C and allowed to differentiate using the same differentiation culture conditions described above. For chemical chaperone treatment, the EBs were treated with 200 μM tauroursodeoxycholic acid (TUDCA; Calbiochem) in differentiation culture medium for 12 days; the medium was changed every 2 days.

### 2.4. RNA Isolation and cDNA Synthesis

Total RNA was extracted from the cultured cells using the easy-BLUE Total RNA Extraction Kit (iNtRon Biotechnology DR, Korea) according to the manufacturer’s instructions. In brief, the cells were treated with 1 mL of easy-BLUE and the resultant cell lysate was passed through a pipette tip several times. Homogenized samples were incubated for 5 min, and then 0.2 mL of chloroform was added to the samples. The tubes were shaken vigorously, incubated at room temperature for 10 min, and centrifuged at 13,000× *g* for 10 min at 4 °C. The aqueous phase was transferred to a new tube, and then 0.5 mL of isopropyl alcohol was added. The samples were centrifuged again at 13,000× *g* for 10 min at 4 °C, and the RNA pellet was washed with 75% ethanol, allowed to air dry, and then dissolved in RNase-free water. The concentration and purity of total RNA were determined using a Nanodrop ND-2000 spectrophotometer (ThermoFisher Scientific, Wilmington, DE, USA). One microgram of total RNA was transcribed into complementary DNA (cDNA) using All-in-one 5× first strand cDNA Synthesis Master Mix (Cellsafe, Korea).

### 2.5. Reverse Transcription-Quantitative Polymerase Chain Reaction (RT-qPCR)

The synthesized cDNA was used as a template for qPCR with rTaq plus 5× PCR Master Mix (Elpisbio), and the PCR products were detected by 1.2% agarose gel electrophoresis. The relative mRNA levels were calculated from the comparative threshold cycle (Ct) values relative to *GAPDH* levels using the CFX96 Real-Time PCR detection system (Bio-Rad, Hercules, CA, USA) with SYBR green PCR Master Mix (Applied Biosystems, Foster City, CA, USA) according to the manufacturer’s instructions. The results were acquired by comparison with normally conditioned EBs. The target genes were amplified with the primers listed in Table 1.

### 2.6. RT2 Profiler PCR Array and TaqMan hPSC Scorecard Assay

To identify the expression levels of heat shock protein-coding genes that were altered under hyperthermia culture conditions, we used the Human Heat Shock Proteins & Chaperones RT^2^ PCR Array (Qiagen, Germany). Synthesized cDNA was diluted with nuclease-free water and added to the SYBR green Master Mix. Then, 25 μL mixture was added to each well of the PCR array, and real-time PCR was performed on the StepOne Real-Time PCR system (Applied Biosystems). SYBR green fluorescence detection was used with the following thermal profile: initial denaturation at 95 °C for 10 min, followed by 40 cycles of denaturation at 95 °C for 15 s and annealing at 60 °C for 1 min. All PCR data were collected and analyzed using the online platform provided by Qiagen [19].

The TaqMan hPSC Scorecard was used to assess the tri-lineage differentiation potential of cells under different conditions. Scorecard assay analysis was performed using a TaqMan hPSC Scorecard Panel 2× 96w FAST kit (A15871; Applied Biosystems) per the manufacturer’s protocol [20,21]. SYBR green detection was used with the following thermal profile: initial denaturation at 95 °C for 20 s, followed by 40 cycles of denaturation at 95 °C for 1 s and annealing at 60 °C for 20 s. Comparisons were made among cells in the normal group (37 °C), hyperthermia group (42 °C), and group treated with tauroursodeoxycholic acid (TUDCA) (200 μM). The gene expression profiles of each group were compared with a common gene set of reference data using the analysis software provided by Thermo Fisher Scientific.

### 2.7. Statistical Analysis

All experiments were performed three times in triplicate and the data are represented as mean ± SD for statistical comparisons. The statistical significance of the differences between the groups was evaluated using the Student’s *t* test. The results with *p* values less than 0.05 were considered statistically significant.

## 3. Results

### 3.1. Optimizing the Conditions of Hyperthermia

**Figure 1:**
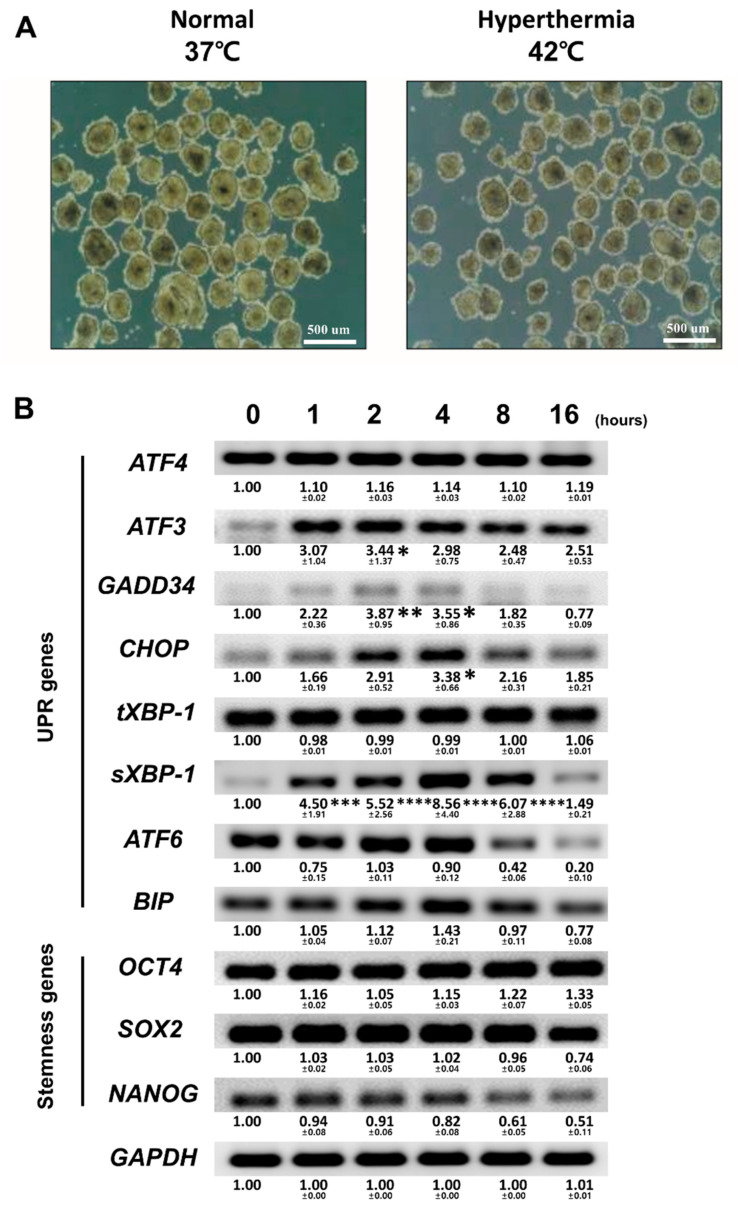
Determination of the optimal experimental hyperthermic conditions for unfolded protein response (UPR) activation without causing human embryonic stem cell (hESC) death. (**A**) Phase-contrast images showing in vitro spontaneous differentiation of the hESCs under normal and hyperthermic conditions. (**B**) Gene expression analysis by RT-qPCR using total RNA isolated from spontaneously differentiated EBs exposed to a hyperthermic condition for the indicated times. The embryoid bodies (EBs) expressed UPR genes and stemness genes. Fold changes of signal intensity were normalized by *GAPDH* intensities. Fold changes represent the mean values obtained from the three independent experiments. Statistical significance was determined by comparing the relative band intensities referring to those of undifferentiated hESCs as 1. Student’s *t*-test: * *p* < 0.05, ** *p* < 0.01, and *** *p* < 0.001, **** *p* < 0.0001.

To determine the optimal exposure time to hyperthermia for this in vitro model, we first compared the induction of UPR-related genes under exposure to 42 °C for different lengths of time while maintaining EB formation without causing hESC death. The hESC colonies were manually dissected and cultured in suspension for 2 days to induce EB formation, and the samples were harvested every hour to analyze mRNA expression levels (Figure 1A,B), whereas EBs were cultured at 42 °C. There was no change in the expression levels of stemness marker genes for the first 16 h under heat stress, whereas the expression level of *NANOG* slightly decreased in EBs exposed to hyperthermic conditions for 8 and 16 h. Meanwhile, the expression levels of UPR genes *GADD34*, *CHOP, sXBP-1*, and *BIP* were the highest when exposed to heat stress for 4 h. The expression levels of most UPR-related genes returned to normal when cultured for longer periods under hyperthermia. Therefore, culturing hESCs for 4 h at 42 °C was the most suitable exposure period to obtain the highest expression level of UPR-related genes while minimizing stress-mediated cell death.

### 3.2. Hyperthermia Disturbs Spontaneously Differentiating hEBs through UPR Induction

**Figure 2:**
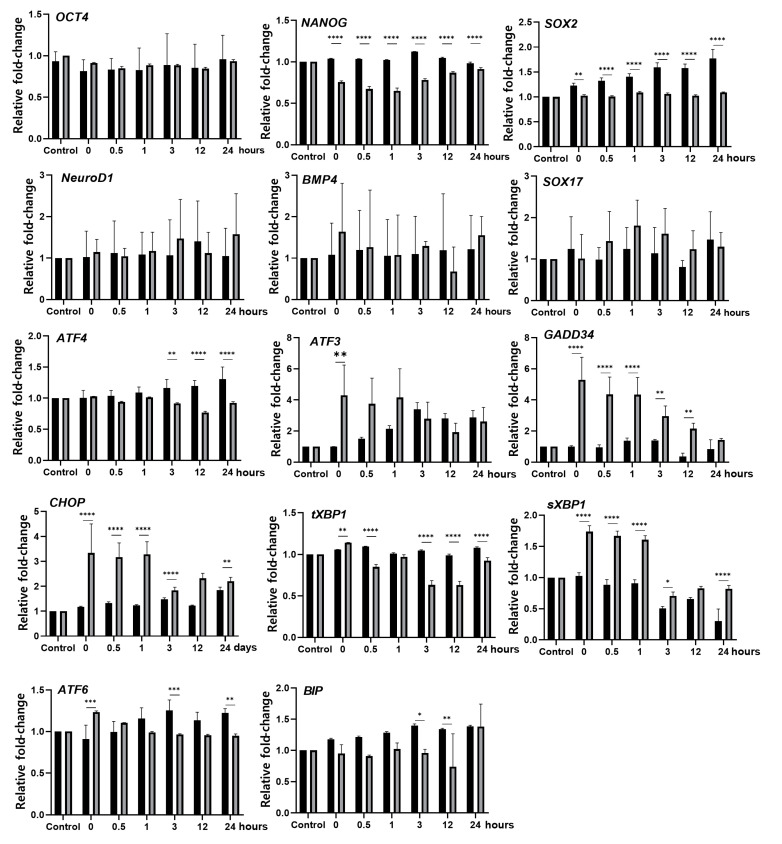
Hyperthermia activates the embryoid body (EB) stress pathway in human EBs. RT-qPCR analysis of the EBs cultured under normothermic (black bars) and hyperthermic (gray bars) conditions. Gene expression of the markers of stemness (*OCT4, NANOG*, and *SOX2*), lineage-specific markers (*NeuroD1*, *BMP4*, and *SOX17*), and ER stress-induced UPR-related genes (*ATF4, ATF3, GADD34, CHOP, tXBP1, sXBP1, ATF6*, and *BIP*). The housekeeping gene *GAPDH* was used as a loading control. Fold changes of signal intensity were normalized by *GAPDH* intensities. Fold changes shown are the mean values obtained from three independent experiments. Statistical significance was determined by comparing the relative band intensities and referring to those of undifferentiated hESCs as 1. Student’s *t*-test: * *p* < 0.05, ** *p* < 0.01, and *** *p* < 0.001, **** *p* < 0.0001.

EBs cultured for 4 h at 37 °C (normothermia) and 42 °C (hyperthermia) were then induced to spontaneously differentiate, and the gene expression levels were examined at 0–24 h. In both temperature groups, no significant difference was observed in the expression levels of stemness marker genes (*OCT4, NANOG*, and *SOX2*) and the differentiation marker genes (*NeuroD1*, *BMP4,* and *SOX17*) over time (Figure 2 and Supplemental Figure S1). Although the expression of the stemness marker genes *NANOG* and *SOX2* was significantly decreased by the hyperthermia condition compared to normothermia, the differentiation marker genes did not change during this incubation period as it was likely too narrow for any changes in gene expression to occur. By contrast, the expression levels of the UPR-related genes *CHOP, ATF3*, and *GADD34* continued to increase for up to 1 h of differentiation in EBs cultured under hyperthermia, and then declined gradually over time (Figure 2 and Supplemental Figure S1). The expression levels of other UPR-related genes (*ATF4* and *ATF6*) were not affected by heat stress.

**Figure 3:**
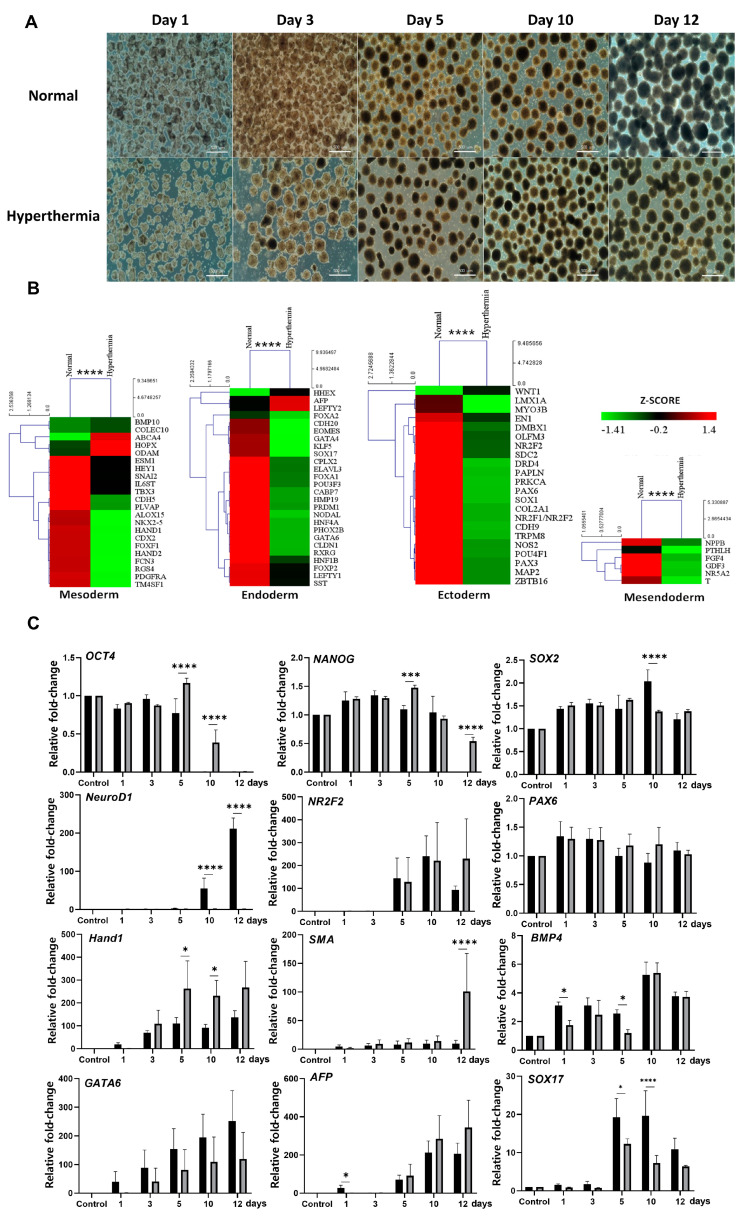
Hyperthermia disturbs spontaneously differentiating human EBs (hEBs) through UPR induction. (**A**) Phase-contrast images showing the in vitro spontaneous differentiation of the hESCs for the indicated days after exposure to hyperthermia or normothermia for 4 h. (**B**) Heatmap of the differentiation potential of all the gene classes (mesoderm, endoderm, ectoderm, mesendoderm, and self-renewal) of EBs at day 12 after exposure to hyperthermia or normothermia for 4 h. (**C**) Expression of pluripotency markers (*OCT4, NANOG*, and *SOX2*) and differentiation markers (ectoderm: *NeuroD1, NR2F2, PAX6*; mesoderm: *Hand1, SMA, BMP4*; endoderm: *GATA6, AFP, SOX17*) examined by RT-qPCR. The black bars indicate the normal condition and the gray bars indicate the hyperthermia condition. The housekeeping gene *GAPDH* was used as a loading control. Fold changes in the signal intensity were normalized with *GAPDH*. Fold changes shown are the mean values obtained from three independent experiments. Statistical significance was determined by comparing the relative band intensities and referring to those of undifferentiated hESCs as 1. Student’s *t*-test: * *p* < 0.05, ** *p* < 0.01, and *** *p* < 0.001, **** *p* < 0.0001.

The effects of heat stress on the differentiation of hESCs into the three germ layers were examined immediately after exposure to the hyperthermia treatment (Figure 3). The EBs were cultured for 4 h under normothermia and hyperthermia culture conditions, and were then induced to trigger differentiation for 12 days in the same differentiation medium (Figure 3A). The UPR-related genes and genes of different lineages were then compared using the TaqMan hPSC Scorecard analysis and RT-qPCR to evaluate the induction of the UPR. These data demonstrated greater the suppression of the expression of the three-germ-layer-associated genes in the hyperthermia group than in the normal temperature group (Figure 3B).

To identify the expression patterns of three-germ-layer genes in response to hyperthermia, we examined the mRNA expression in EBs differentiated for 1, 3, 5, 10, and 12 days. The expression of *OCT4* and *NANOG* was evident in undifferentiated EBs, and apparently suppressed at day 5 after differentiation induction at normal temperature, whereas the expression of *OCT4* and *NANOG* persisted even after day 10 of differentiation in EBs exposed to hyperthermic conditions (Figure 3C and Supplemental Figure S2). The expression of three-germ-layer genes was gradually induced in suspended EB cultures, and the EBs exposed to hyperthermic conditions showed distinct gene expression patterns from those differentiating under normal physiological temperature. Specifically, the expression of mesoderm genes *Hand1* and *SMA* was observed from day 3 and day 1 of differentiation of EBs exposed to hyperthermia or a normal temperature, respectively. Expression levels of the ectoderm gene *NeuroD1* in EBs differentiated under hyperthermic conditions remained very low until day 12. The levels of endoderm genes (*GATA6*, *AFP,* and *SOX17*) and the ectoderm gene *NR2F2* in EBs were similar under hyperthermia and normal conditions. These results suggest that hyperthermia stress disturbs the process of hESC differentiation.

### 3.3. TUDCA Suppresses the Expression of UPR-Related Genes

**Figure 4:**
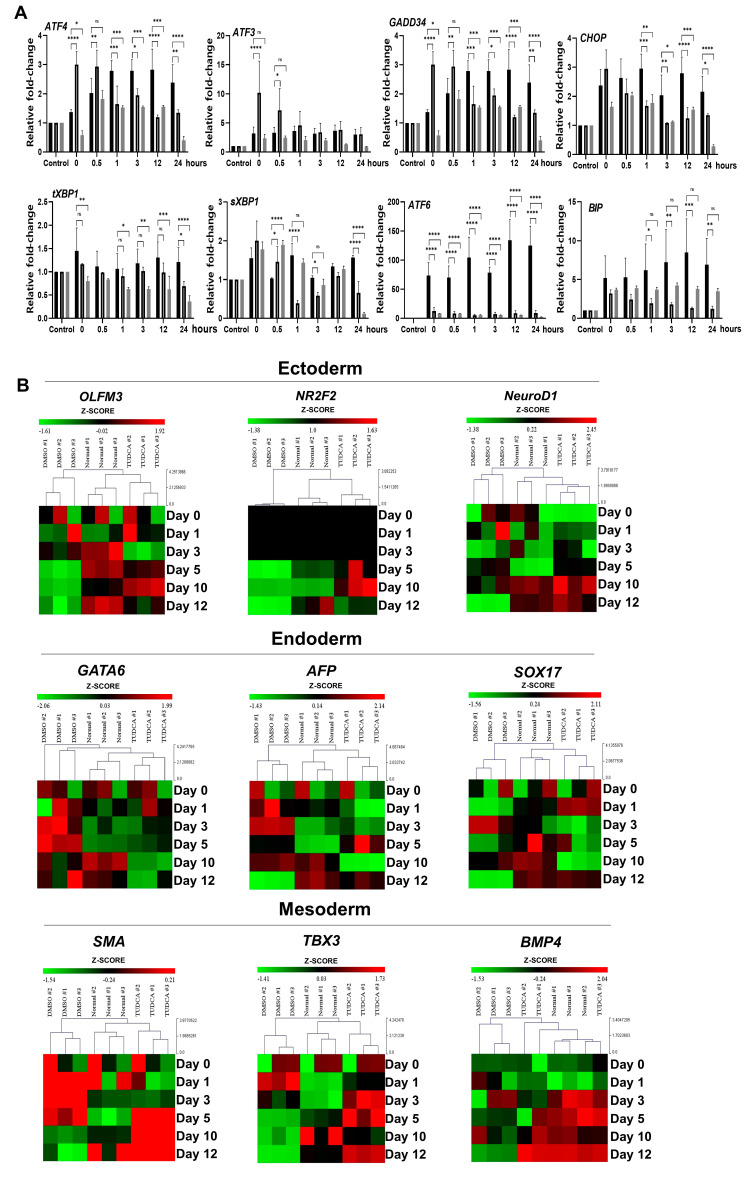
Tauroursodeoxycholic acid (TUDCA) suppressed the induction of UPR-related genes under heat stress. (**A**) UPR-related genes measured by RT-qPCR. Left: EBs were cultured under normal conditions for the indicated times; right: DMSO (control)- or TUDCA-treated EBs cultured under hyperthermic conditions for the indicated times. Fold changes shown are the mean values obtained from three independent experiments. Statistical significance was determined by comparing the relative band intensities with those of undifferentiated hESCs as 1. Student’s *t*-test: * *p* < 0.05, ** *p* < 0.01, and *** *p* < 0.001, **** *p* < 0.0001. The Black bars indicate the normal condition, black border gray bars indicate the hyperthermic condition, and gray bars indicate the hyperthermic condition with TUDCA. (**B**) Heatmap of the differentiation markers (ectoderm: *OLFM3, NR2F2, NeuroD1;* mesoderm: *SMA, TBX3, BMP4;* endoderm: *GATA6, AFP, SOX17*) expressed over time from day 0 to day 12.

To identify whether the heat stress-induced disruption of hESC differentiation was regulated by ER stress, we treated the EBs with the chemical chaperone TUDCA, and examined the influence on UPR-related genes and differentiation-related genes (Figure 4A and Supplemental Figure S3). After EB formation, we divided the EBs into groups grown under normal conditions, cultured for 4 h under the hyperthermia condition, and treated with TUDCA after hyperthermia treatment. We then examined the UPR-related gene expression every hour. Compared with the group differentiated under normal conditions, the hyperthermia condition significantly increased the expression levels of *ATF3, GADD34, sXBP1*, and *ATF6* in a shorter period of time, whereas TUDCA suppressed the increase in UPR-related genes, decreasing their expression to near-basal levels.

In the early hours following the exposure of EBs to hyperthermia, the induction of UPR-related expression was disturbed or the hESC differentiation into three germ layers was delayed. In TUDCA-treated EBs exposed to heat stress, we determined the effect of reduced ER stress on early hESC differentiation by analyzing the expression of three-germ-layer-specific genes such as *OLFM3*, *NR2F2, NeuroD1, GATA6*, *AFP, SOX17*, *SMA, TBX3* and *BMP4* with RT-qPCR (Figure 4B). We obtained the z-score for the expression of three-germ-layer-specific genes and the similarity among the three groups (normal, hyperthermia, and TUDCA + hyperthermia) from hierarchical clustering. Figure 4B shows the expression levels of ectoderm-specific genes (*OLFM3*, *NR2F2* and *NeuroD1*), mesoderm-specific genes (*SMA, TBX3* and *BMP4*), endoderm-specific genes (*GATA6, AFP* and *SOX17*). Expression levels of mesoderm-specific genes were generally lower under hyperthermic conditions compared with those detected under normal temperature. However, the TUDCA treatment recovered the expression of these genes to the level observed under normal temperature. Changes in the expression of ectoderm- and endoderm-specific genes by hyperthermia were as notable as those of mesoderm-specific genes, but there was only a marginal effect of TUDCA on ectoderm and endoderm genes.

## 4. Discussion

The present study provides new insights to reveal that hyperthermia disturbs ER homeostasis in hESC differentiation and induces UPR-related gene expression, which may have a significant effect on early embryonic development. In particular, treatment with TUDCA, which is known to attenuate ER stress and prevent UPR dysfunction in differentiating hESCs exposed to heat stress, suppressed the induction of UPR-related genes and restored the expression of organ differentiation-related genes to normal levels [22]. Hence, the present model could effectively recapitulate early human embryo differentiation in vitro using hESCs, revealing that the abnormal differentiation caused by heat stress is mediated by the ER stress response.

A human embryo or fetus is precisely programmed to differentiate and develop into a complete individual. During pregnancy, from fertilization to birth, an embryo or fetus responds to continuous extrinsic and intrinsic stimuli, undergoes normal gene transcription and translation, which tightly regulate cell proliferation, migration, and apoptosis [23]. In addition to the regulators involved in normal embryogenesis, an embryo or fetus is exposed to a variety of cell stress stimuli, which are recognized as important elements in normal embryogenesis.

In general, if exposed to relatively mild heat stress before an embryo is implanted to the uterine wall, no abnormality occurs during the ensuing embryonic development; in contrast, if continuously exposed to heat stress to an extent that causes damage to the embryo, the embryo tends to not be implanted [11]. After implantation, an embryo enters a period of rapid cell proliferation to develop organs after organogenesis. This is the period in which the embryo is most susceptible to hyperthermia, and in which stress exposure may cause various developmental malformations. A developing brain or central nervous system seems to be particularly vulnerable under hyperthermic conditions, which can lead to craniofacial, skeletal, and heart malformations [1]. The earlier the damage inflicted to a developing embryo, the more severe abnormal development becomes, with more organs ultimately being affected. Heat stress exposure in later fetal development stages mostly delays growth, but does not have the same effect as that in early embryonic development [1]. Since a developing embryo has limited capacity to control temperature on its own, the embryo entirely relies on the mother for temperature control. Radiation, diseases, and viral infections are common heat stressors during pregnancy [13]. However, recent studies have also identified increasing temperatures caused by global warming as another heat stressor. According to the Ghana maternal health survey, exposure to high heat is harmful to pregnancy, and as the global temperature increases, stillbirths or miscarriages also increase, although this finding is not statistically significant [24]. In addition, a Canadian clinical cohort study found that prolonged exposure to over 30 °C can possibly cause non-critical congenital heart defects, such as atrial septal defects [25]. However, as each animal model has different temperature thresholds, our current knowledge is limited to the effects of hyperthermia from models of malformations confirmed in different experimental animals. Hence, it is important to establish a model that can replicate human embryogenesis in vitro. In this respect, we have demonstrated that hESCs can be used as a cell source to best represent human embryogenesis.

In general, cells exposed to hyperthermia respond with a cascade of molecular mechanisms associated with human stem cells. The occurrence of severe heat-related adverse effects in embryogenesis hinders normal embryogenesis due to changes in the physical structure of intracellular proteins (especially enzymes) with a consequent loss of enzyme activity [13]. However, detailed studies on ER stress response mechanisms caused by heat exposure are scarce. Hence, we focused on the ER stress response mechanism in developing hESCs exposed to hyperthermia involving denatured and unfolded proteins. Since differentiating hESCs have a shorter cell cycle, they also have less time to recover from extracellular stress, making them more vulnerable to heat stress. Moreover, many unfolded proteins accumulate under heat stress. To regulate the accumulated unfolded proteins in the cells, UPR-related genes are induced to maintain protein homeostasis. Three UPR signaling pathways are mediated by three signaling systems in the ER: PERK, IRE-1a, and ATF6. In our study, only the levels of PERK signaling-related genes were increased by heat stress in hESCs, and there was almost no change in the expression of downstream genes related to the IRE-1a and ATF6 signaling pathways. PERK, a type I transmembrane protein kinase, is activated through autophosphorylation under elevated ER stress, and subsequently phosphorylates eukaryotic initiation factor 2 alpha subunit (eIF2α) to inhibit general transcription and translation processes. Phosphorylated eIF2α inhibits the formation of a complex that is important for initiating protein translation by inhibiting the activity of eIF2B, thereby reducing the amount of unfolded proteins entering the ER. Conversely, the translation of *ATF4*, which is inactivated under normal conditions, increases protein translation due to eIF2α phosphorylation, thereby increasing the transcription of *GADD34* to regulate protein synthesis; alternatively, ATF4 also induces apoptosis by increasing the transcription of *CHOP*. As shown in Figure 2, differentiating EBs exposed to hyperthermia appear to regulate the amount of unfolded proteins entering the ER by reducing the expression of differentiation-related genes. In addition, the treatment of cells with the chemical chaperone TUDCA significantly reduced PERK signaling, which was increased in a short period of time under heat stress, and restored the levels of three-germ-layer-specific genes to normal. This finding is consistent with that of a previous study, which showed that relieving ER stress in mammalian cells in vitro had positive effects on embryo survival and development [26]. However, how TUDCA affects embryonic development and protects embryos from heat stress using an in vivo model should be explored in the future.

In conclusion, the present study demonstrates that heat stress disturbs the early differentiation of hESCs associated with ER stress, and provides a new perspective on how hESCs control and respond to external stresses such as hyperthermia.

## Figures and Tables

**Table 1:** The sequence of primers used in RT-qPCR.

Primers	Sequences	Length (bp)	Annealing Temperature (°C)
*GAPDH*	F: 5’- GGT GTG AAC CAT GAG AAG TAT GA-3’	123	60
	R: 5 - GAG TCC TTC CAC GAT ACC AAA G-3’		
*OCT4*	F: 5’- TAT GGG AGC CCT CAC TTC AC-3’	144	60
	R: 5’- AGT TTG TGC CAG GGT TTT TG -3’		
*SOX2*	F: 5′- GCT AGT CTC CAA GCG ACG AA -3′	144	60
	R: 5′- GCA AGA AGC CTC TCC TTG AA -3′		
*NANOG*	F: 5′- CAG TCT GGA CAC TGG CTG AA -3′	149	60
	R: 5′- CTC GCT GAT TAG GCT CCA AC -3′		
*BMP4*	F: 5′- GGA GAT GGT AGT AGA GGG ATG T -3′	105	60
	R: 5′- CGT GTG TGT GTG GTG TAT GT -3′		
*SOX17*	F: 5′- CAT GAC TCC GGT GTG AAT CTC -3′	99	60
	R: 5′- CAC GTC AGG ATA GTT GCA GTA ATA -3′		
*ATF3*	F: 5’- CCT CTG CGC TGG AAT CAG TC-3’	111	60
	R: 5’- TTC TTT CTC GTC GCC TCT TTT T-3’		
*ATF4*	F: 5’- ATG ACC GAA ATG AGC TTC CTG-3’	153	60
	R: 5’- GCT GGA GAA CCC ATG AGG T-3’		
*ATF6*	F: 5’- TCC TCG GTC AGT GGA CTC TTA-3’	235	60
	R: 5’- CTT GGG CTG AAT TGA AGG TTT TG-3’		
*CHOP*	F: 5’- GGA AAC AGA GTG GTC ATT CCC-3’	116	60
	R: 5’- CTG CTT GAG CCG TTC ATT CTC-3’		
*BIP*	F: 5’- CAT CAC GCC GTC CTA TGT CG-3′	104	60
	R: 5′- CGT CAA AGA CCG TGT TCT CG-3′		
*GADD34*	F: 5′- ATG ATG GCA TGT ATG GTG AGC-3′	120	60
	R: 5′- AAC CTT GCA GTG TCC TTA TCA G-3′		
*sXBP-1*	F: 5′- GGT CTG CTG AGT CCG CAG CAG G-3′	311	60
	R: 5′- GGG CTT GGT ATA TAT GTG G-3′		
*tXBP-1*	F: 5′- CCC TCC AGA ACA TCT CCC CAT-3′	101	60
	R: 5′- ACA TGA CTG GGT CCA AGT TGT-3′		
*NEUROD1*	F: 5’- GAA CGC AGA GGA GGA CTC AC-3’	109	60
	R: 5’- CTT GGG CTT TTG ATC GTC AT-3’		
*TBX3*	F: 5’- GAT GAG TCC TCC AGT GAA CAA G-3’	91	60
	R: 5’- CTT TGA GGT TCG ATG TCC CTA C-3’		
*Hand1*	F: 5’- CGC CTA GCC ACC AGC TAC ATC-3’	106	60
	R: 5’- CGC CAT CCG CCT TCT TGA GTT-3’		
*SMA*	F: 5’- ACC CAC AAT GTC CCC ATC TA-3’	123	60
	R: 5’- GAA GGA ATA GCC ACG CTC AG-3’		
*GATA6*	F: 5’- TCC ACT CGT GTC TGC TTT TG-3’	140	60
	R: 5’- CCC TTC CCT TCC ATC TTC TC-3’		
*AFP*	F: 5’- AAA TGC GTT TCT CGT TGC TT-3’	136	60
	R: 5’- GCC ACA GGC CAA TAG TTT GT-3’		
*OFLM3*	F: 5′- ACC CAG TTC AAG GAG GAA ATA AG-3′	104	60
	R: 5′- CTC AGC ACT CTT TGG TGT AGT T-3′		
*NR2F2*	F: 5′- GAA GAT CTC TCC CTT CAC CTT TC-3′	100	60
	R: 5′- GAA TCT CCT CCT CGG TGT TTA TC-3′		

## Supplementary Materials

The following are available online at https://www.mdpi.com/2227-9059/8/6/176/s1.
