# Supplementary material for: Hyperthermia Disturbs and Delays Spontaneous Differentiation of Human Embryoid Bodies
Source: Biomedicines. 2020 Jun 26;8(6):176. doi: 10.3390/biomedicines8060176 (PMC7345654; doi:10.3390/biomedicines8060176)
Supplement: Supplementary file 1 [file biomedicines-08-00176-s001.pdf]

Supplemental figure 1.

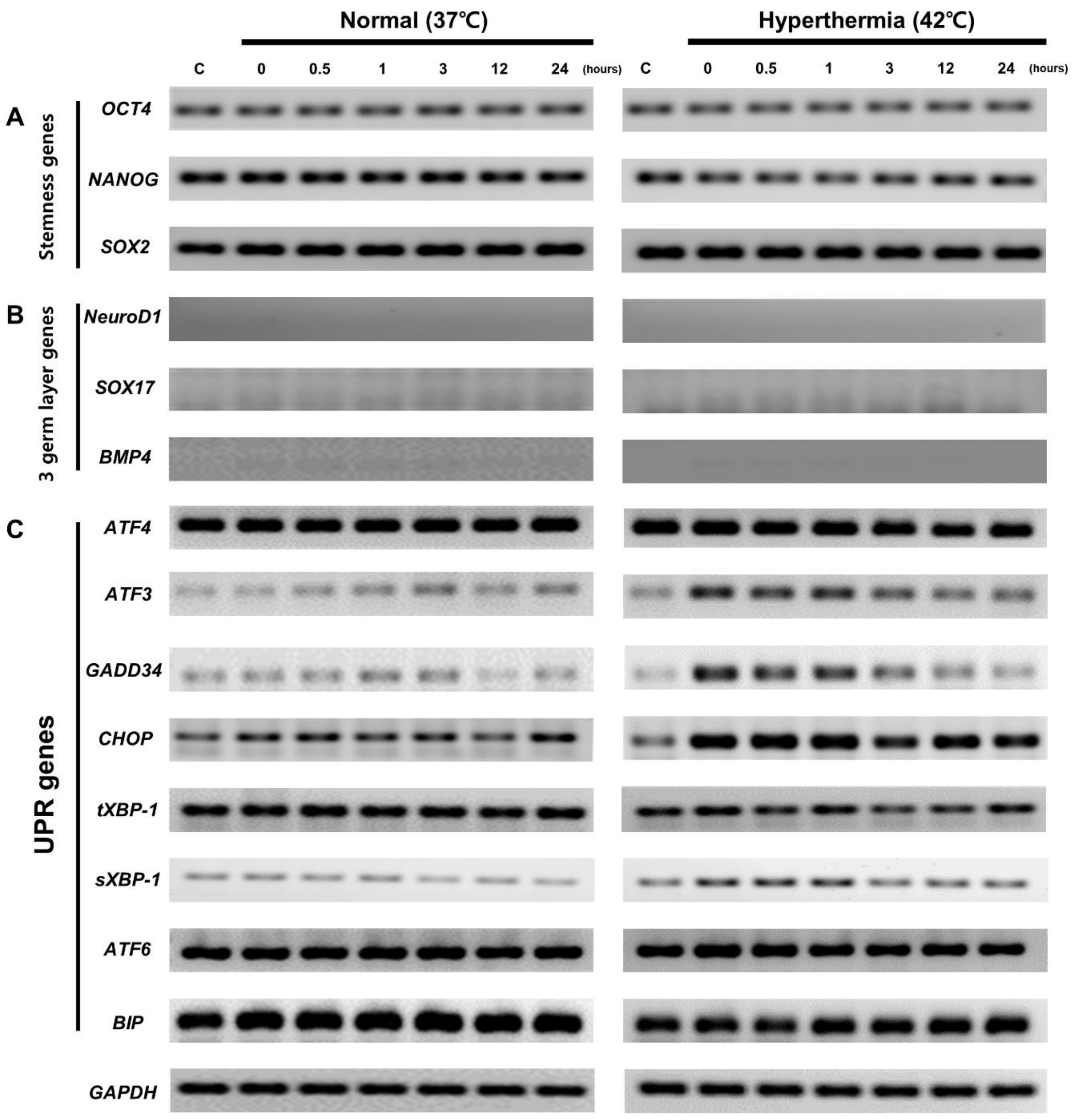

Supplemental figure 1. Hyperthermia activates the ER stress pathway in human EBs. RT-qPCR analysis of EBs cultured under normal and hyperthermic conditions. (**A**) Gene expression of markers of stemness (*OCT4, NANOG*, and *SOX2*), (**B**) lineage-specific markers (*NeuroD1, BMP4* and *SOX17*), and (**C**) ER stress-induced UPR-ralated genes (*ATF4, ATF3, GADD34, CHOP, tXBP1, sXBP1, ATF6*, and *BIP*). The housekeeping gene *GAPDH* was used as loading control. Fold changes of signal intensity were normalized by *GAPDH* intensities.

Supplemental figure 2.

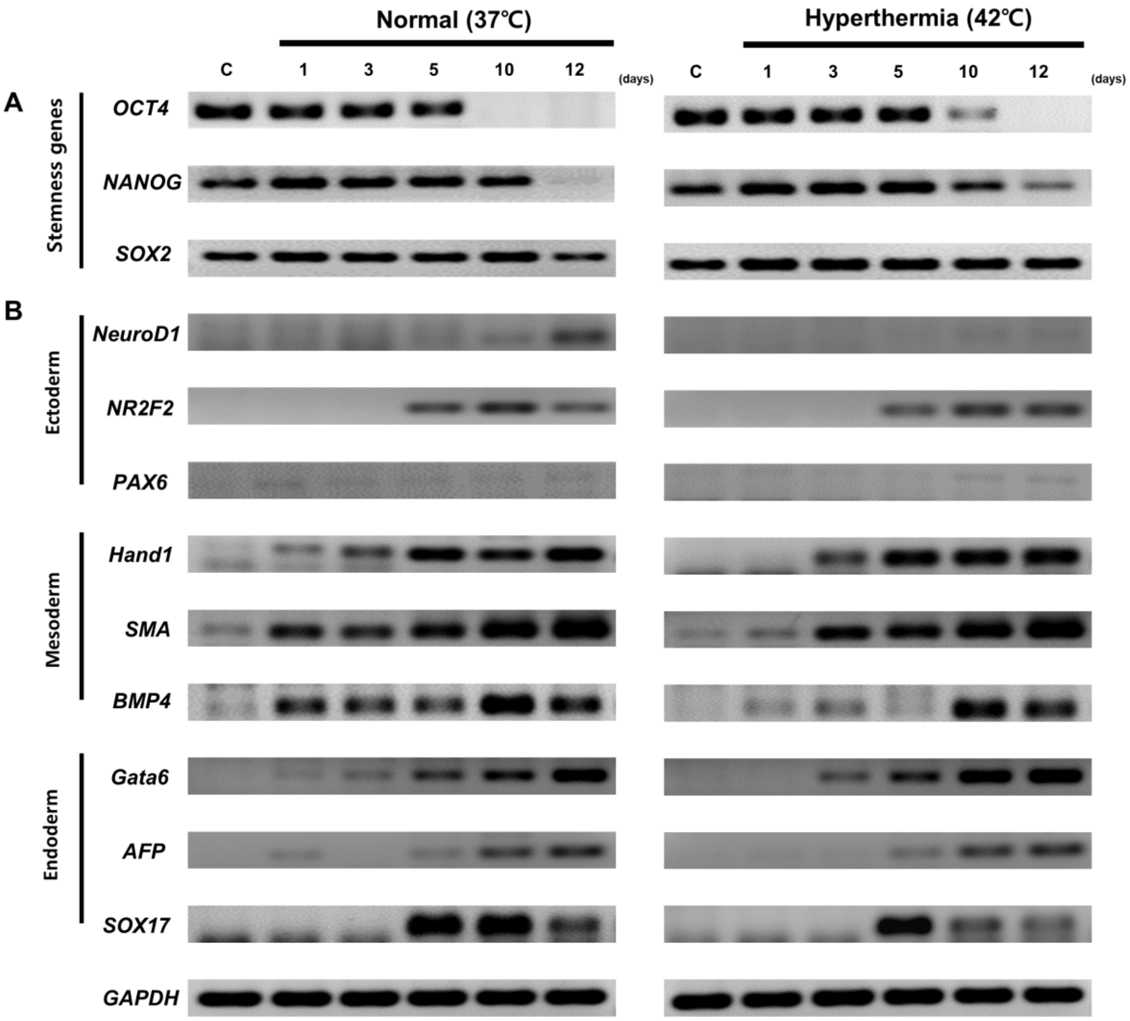

Supplemental figure 2. Hyperthermia disturbs spontaneously differentiating hEBs through UPR induction. (**A, and B**) Expression of pluripotency markers (*OCT4, NANOG*, and *SOX2*) and differentiation markers (ectoderm: *NeuroD1, NR2F2, PAX6*; mesoderm: *HAND1, SMA, BMP4*; endoderm: *GATA6, AFP, SOX17*) examined by RT-qPCR. The housekeeping gene (*GAPDH*) was used as a loading control.

Supplemental figure 3.

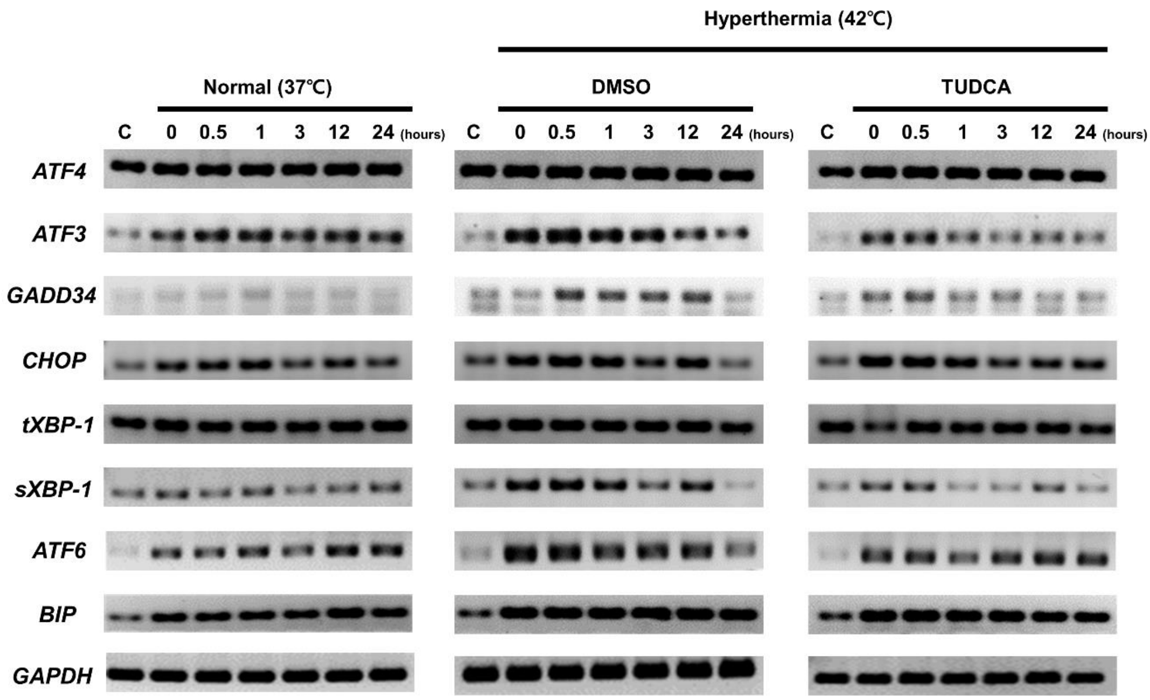

Supplemental figure 3. TUDCA suppressed the induction of UPR-related genes under heat stress. UPR-related genes measured by RT-qPCR. Left: EBs were cultured under normal conditions for the indicated times; right: DMSO (control)- or TUDCA-treated EBs cultured under hyperthermic conditions for the indicated times.
